# Fibroblast Growth Factor 1 Ameliorates Diabetes-Induced Liver Injury by Reducing Cellular Stress and Restoring Autophagy

**DOI:** 10.3389/fphar.2020.00052

**Published:** 2020-03-03

**Authors:** Zeping Xu, Yanqing Wu, Fan Wang, Xiaofeng Li, Ping Wang, Yuying Li, Junnan Wu, Yiyang Li, Ting Jiang, Xindian Pan, Xie Zhang, Longteng Xie, Jian Xiao, Yanlong Liu

**Affiliations:** ^1^ School of Pharmaceutical Sciences, Wenzhou Medical University, Wenzhou, China; ^2^ Institute of Life Sciences, Wenzhou University, Wenzhou, China; ^3^ The Second Affiliated Hospital, Xinjiang Medical University, Urumqi, China; ^4^ Beijing Hui-Long-Guan Hospital, Peking University, Beijing, China; ^5^ School of Medicine, Hangzhou Normal University, Hangzhou, China; ^6^ Department of Pharmacy, Ningbo Medical Treatment Center, Li Huili Hospital, Ningbo, China; ^7^ Department of Infection Diseases, Ningbo Fourth Hospital, Xiangshan, China; ^8^ Center for Health Assessment, Wenzhou Medical University, Wenzhou, China

**Keywords:** fibroblast growth factor 1, diabetes, liver injury, oxidative stress, endoplasmic reticulum stress

## Abstract

**Background:**

Type 2 diabetes (T2D) is a metabolic dysfunction disease that causes several complications. Liver injury is one of these that severely affects patients with diabetes. Fibroblast growth factor 1 (FGF1) has glucose-lowering activity and plays a role in modulation of several liver injuries. Nevertheless, the effects and potential mechanisms of FGF1 against diabetes-induced liver injury are unknown.

**Methods:**

To further investigate the effect of FGF1 on diabetic liver injury, we divided db/db mice into two groups and intraperitoneally (i.p.) injected either with FGF1 at 0.5 mg/kg body weight or saline every other day for 4 weeks. Then body weights were measured. Serum and liver tissues were collected for biochemical and molecular analyses.

**Results:**

FGF1 significantly reduced blood glucose and ameliorated diabetes-induced liver steatosis, fibrosis, and apoptosis. FGF1 also restored defective hepatic autophagy in db/db mice. Mechanistic investigations showed that diabetes markedly induced oxidative stress and endoplasmic reticulum stress and that FGF1 treatment significantly attenuated these effects.

**Conclusions:**

FGF1-associated glucose level reduction and amelioration of cellular stress are potential protective effects of FGF1 against diabetes-induced liver injury.

## Introduction

Type 2 diabetes (T2D) is a complex metabolic disease characterized by insulin resistance and pancreatic cell failure (Kasuga, 2006). Hyperglycemia resulting in glucotoxicity is the primary pathophysiological trigger of diabetes-induced complications (Wu et al., 2015a; Wu et al., 2016). Liver injury occurs commonly in patients with diabetes (Gezginci-Oktayoglu et al., 2009). Liver damage caused by diabetes is characterized by abnormal liver enzyme levels, steatosis, fibrosis, and cirrhosis. Diabetes-induced liver damage produces several pathological changes in the morphology and microstructure of hepatic tissues and functions, including vacuolization and lipid accumulation, resulting in significant damage (Wu et al., 2015a). There are many mechanisms mediating liver damage caused by diabetes, including hyperglycemia, oxidative stress, endoplasmic reticulum stress, and advanced glycation end-products (JaeMyoung et al., 2014). Therefore, it is important to detail these pathophysiological mechanisms and to explore effective therapeutic strategies to meliorate diabetes-induced liver injury.

Fibroblast growth factor 1 (FGF1) releases from cells through a nonclassical secretory pathway, acting on cells in various tissues, including liver and vasculature, where it exerts classic mitogenic activity (Jiang et al., 2016). FGF1 has an unique ability to lower blood glucose, and pharmacologically-relevant FGF1 (0.5 mg/kg) leads to impressive changes in several measurements, including blood glucose, nearly normalizing after 35 days in T2D mice models with impaired insulin sensitivity (JaeMyoung et al., 2014). Administration of exogenous FGF1 stimulates glucose uptake in an insulin-dependent fashion and suppressed the hepatic production of glucose to achieve whole-body insulin sensitization in a mouse model of T2D (JaeMyoung et al., 2014). As an insulin sensitizer, FGF1 mediates homeostatic control of glycemia by acting on several pathways (Li, 2019). The glucose-lowering activity of FGF1 is dissociated from its mitogenic activity and is mediated predominantly *via* FGF receptor 1 signaling (JaeMyoung et al., 2014).

FGF1 also reverses hepatic lipid steatosis by improving lipid catabolism (Liu W. et al., 2016). Chronic treatment with rFGF1 led to reduced hepatic steatosis and augmented liver glycogen content (JaeMyoung et al., 2014). A recent study showed that FGF1 meliorated acetaminophen (APAP)-induced hepatotoxicity in mice through suppression of inflammation, apoptosis, and oxidative and endoplasmic reticulum stress (Wang et al., 2019). These findings suggest that FGF1 may have hepato-protective properties beyond its anti-steatotic and anti-inflammatory properties. Based on these studies, we aimed to investigate the effects and molecular mechanisms of FGF1 in diabetic-induced liver injury.

## Materials and Methods

### Animal Experiments

Twelve-week-old male db/db mice and their nondiabetic db/m littermates were purchased from the Model Animal Research Center of Nanjing University (Nanjing, China). Animals were maintained under 12:12 h light:dark cycle conditions. The db/db mice were divided into two groups and were intraperitoneally (i.p.) injected either with FGF1 (Guang et al., 2018) (0.5 mg/kg body weight, n = 8) or physiologic saline (n = 8) every other day for 4 weeks (Wu et al., 2018). After 4 weeks, body weights were measured. Then serum and liver tissues samples of these mice were collected for biochemical and molecular analyses.

### Biochemical Analysis

Blood glucose levels were monitored from tail blood samples using a blood glucose meter (Glucometer, SANNUO, China). Serum levels of alanine aminotransferase (ALT), triglyceride (TG), liver glutathione peroxidase (GSH-PX), and liver 4-hydroxynonenal (4-HNE) protein adducts were measured according to the manufacturer's instructions (Jian Cheng Biotechnology Co., Ltd. of Nanjing, China). Total antioxidant capacity (T-AOC), malondialdehyde (MDA), and superoxide dismutase (SOD) activity were also measured with various assay kits (Beyotime Biotechnology Corporation, Shanghai, China). Plasma glycosylated hemoglobin (GHbA1c) levels were measured using the Mouse Glycated Hemoglobin A1c (GHbA1c) ELISA Kit (Enzyme-linked Biotechnology Co., Ltd. Shanghai, China).

### Liver Triglyceride Assay

Hepatic triglyceride (TG) levels were determined as described previously (Liu Y. et al., 2016), using the TG reagent (Thermo Fisher Scientific, Middletown, VA).

### Pathology and Immunohistochemical Staining on Liver Tissue

Hematoxylin and eosin (H&E) staining and Masson's trichrome (MT) staining (Solarbio Science & Technology, Beijing, China G1340) were employed to evaluate the characteristics of liver tissues in histological changes and fibrosis. Immunohistochemical staining was used to further verify the deposition of collagen I (collagen I, 1:500; Abcam, Cambridge, UK) in fibrotic liver. The procedure was performed as described previously (Hao et al., 2009).

### Terminal Deoxynucleotidyl Transferase Deoxyuridine Triphosphate Nick End Labeling Assay

Liver sections of 5 μm were stained for terminal deoxynucleotidyl transferase deoxyuridine triphosphate nick end labeling (TUNEL) according to the manufacturer's instruction using the ApopTag Peroxidase In Situ Apoptosis Detection Kit (Chemicon, CA, USA).

### Western Blotting Analysis

Liver tissues were homogenized and determined as described previously (Wang et al., 2019). The blots were incubated with primary antibodies: cleaved caspase-3, Bax, Bcl-2, ATF6, GRP78, Nrf2, SOD2, collagen IA1, ATG5, p62 and LC3 (Abcam, USA), HO-1 (Santa Cruz Biotechnology, CA, USA), CHOP, P-IRE1α, P-eIF2α, P-PERK, PERK, FGFR1, P-AMPK and AMPK (Cell Signaling Technology, Danvers, MA, US), and α-SMA, TGF-β, and GAPDH (Proteintech, China) followed by incubation with their corresponding secondary antibodies: anti-rabbit and anti-mouse (Proteintech, China).

### Statistical Analysis

All data were expressed as mean ± standard error of the mean (SEM). Statistical differences were determined using one-way ANOVA (for comparison of two experimental conditions). Statistical significance was considered at P values < 0.05. Statistical calculations were done using GraphPad Prism 6 (GraphPad Software, Inc., San Diego, USA).

## Results

### FGF1 Treatment Reduced Blood Glucose and Ameliorated Hepatic Steatosis

**Figure 1:**
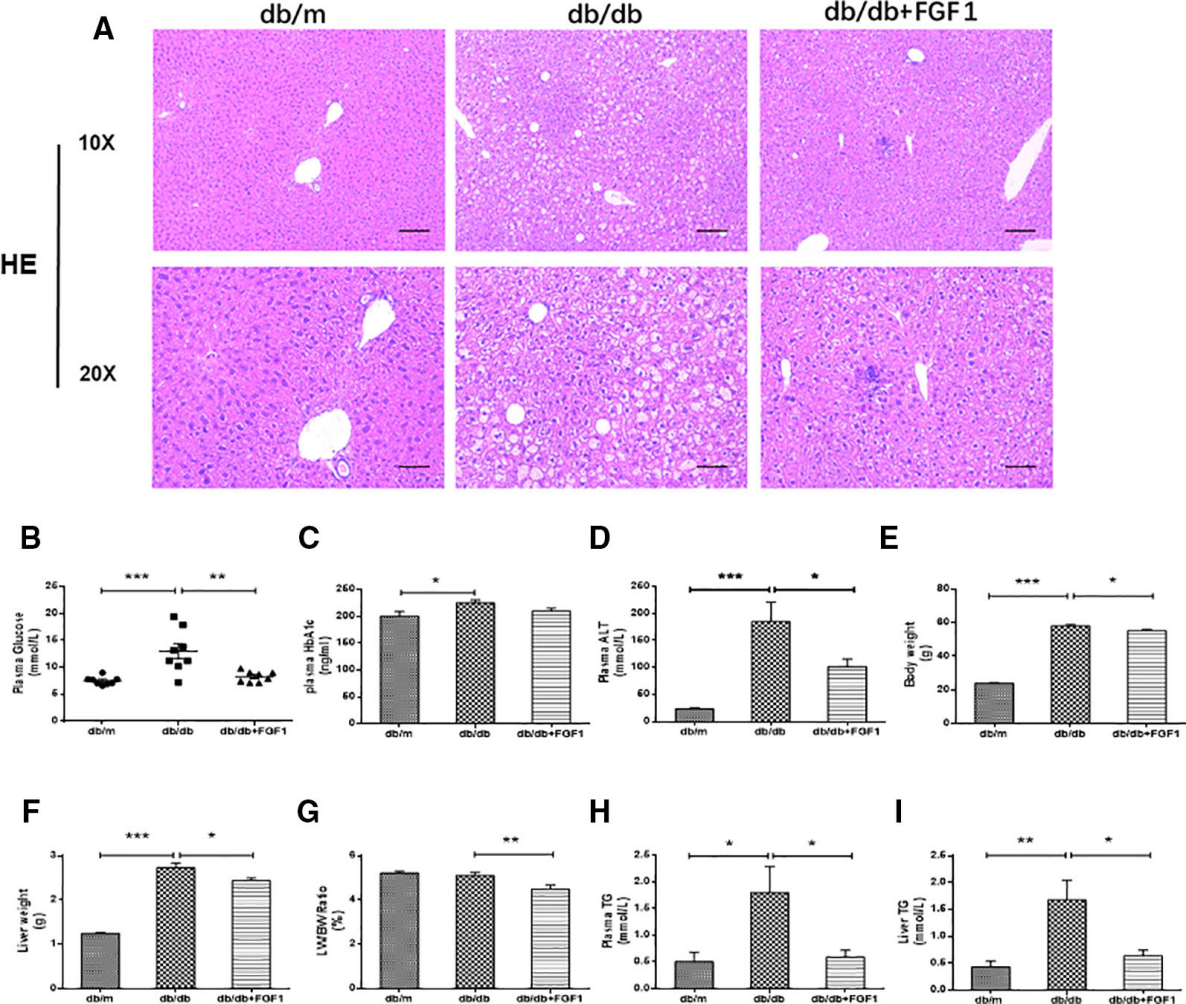
FGF1 ameliorated blood glucose and hepatic steatosis. **(A)** Hematoxylin and eosin (H&E) staining of livers from db/m, db/db, and db/db + FGF1 mice (10 x:scale bars = 100 μm 20 x:scale bars = 50 μm). **(B)** Plasma glucose levels. **(C)** Plasma HbA1C levels. **(D)** Plasma alanine aminotransaminase (ALT) levels. **(E)** Body weights. **(F)** Liver weights. **(G)** Liver weight/body weight ratios. **(H)** Plasma triglyceride (TG) levels. **(I)** Liver TG levels. All data are presented as mean ± SEM, n = 8. *P < 0.05 **P < 0.01 ***P < 0.001 *vs*. the db/m group and db/db + FGF1 group.

A previous study showed that a single injection of FGF1 was sufficient to restore blood glucose levels to the normal range for more than 2 days in both db/db and DIO mouse models (JaeMyoung et al., 2014). In agreement with that study, our results showed that FGF1 treatment markedly reduced blood glucose levels in db/db mice (**Figure 1B**). HbA1c levels in db/db mice were markedly higher than those of db/m mice. No significant differences were found between db/db mice with FGF1 treatment and db/db mice with respect to HbA1c levels (**Figure 1C**). In addition, db/db mice had distinctly elevated plasma alanine aminotransaminase (ALT) levels, a liver injury marker, at 16 weeks of age compared with the db/m mice, an effect that was distinctly reduced by FGF1 treatment (**Figure 1D**). Body weights of db/db mice were significantly greater than those of db/m mice, an effect that was markedly decreased with FGF1 treatment (**Figure 1E**). Compared with db/m mice, liver weights of db/db mice were markedly higher, an effect reversed by FGF1 treatment (**Figure 1F**). Although no significant difference was found between db/m mice and db/db mice with respect to the ratio of liver/body weight, the liver/body weight ratio in db/db mice was reduced by FGF1 treatment (**Figure 1G**). Pathological hepatic lipid accumulation is a characteristic of diabetes liver injury. As previously reported, accumulated fat interferes with insulin signaling pathways and consequently reduces the glucose content taken up from the bloodstream, an effect that is tightly connected to hepatic injury and steatosis in T2DM (Marchesini et al., 2001). In the present study, db/db mice had elevated plasma TG levels, a common feature of dyslipidemia accompanying with type 2 diabetes; this effect was diminished by FGF1 treatment (**Figure 1H**). Histological analyses revealed significant lipid accumulation in liver cells from db/db mice as shown by H&E staining. This effect was significantly attenuated by FGF1 treatment (**Figure 1A**). The db/db mice had elevated liver TG levels, and FGF1 treatment ameliorated these increases (**Figure 1I**). Taken together, our results suggest that db/db mice had fatty livers, and FGF1 treatment reduced blood glucose and attenuated diabetes-induced hepatic steatosis.

### FGF1 Treatment Ameliorated Diabetes-Induced Liver Fibrosis by Reducing Deposition of Collagen

**Figure 2:**
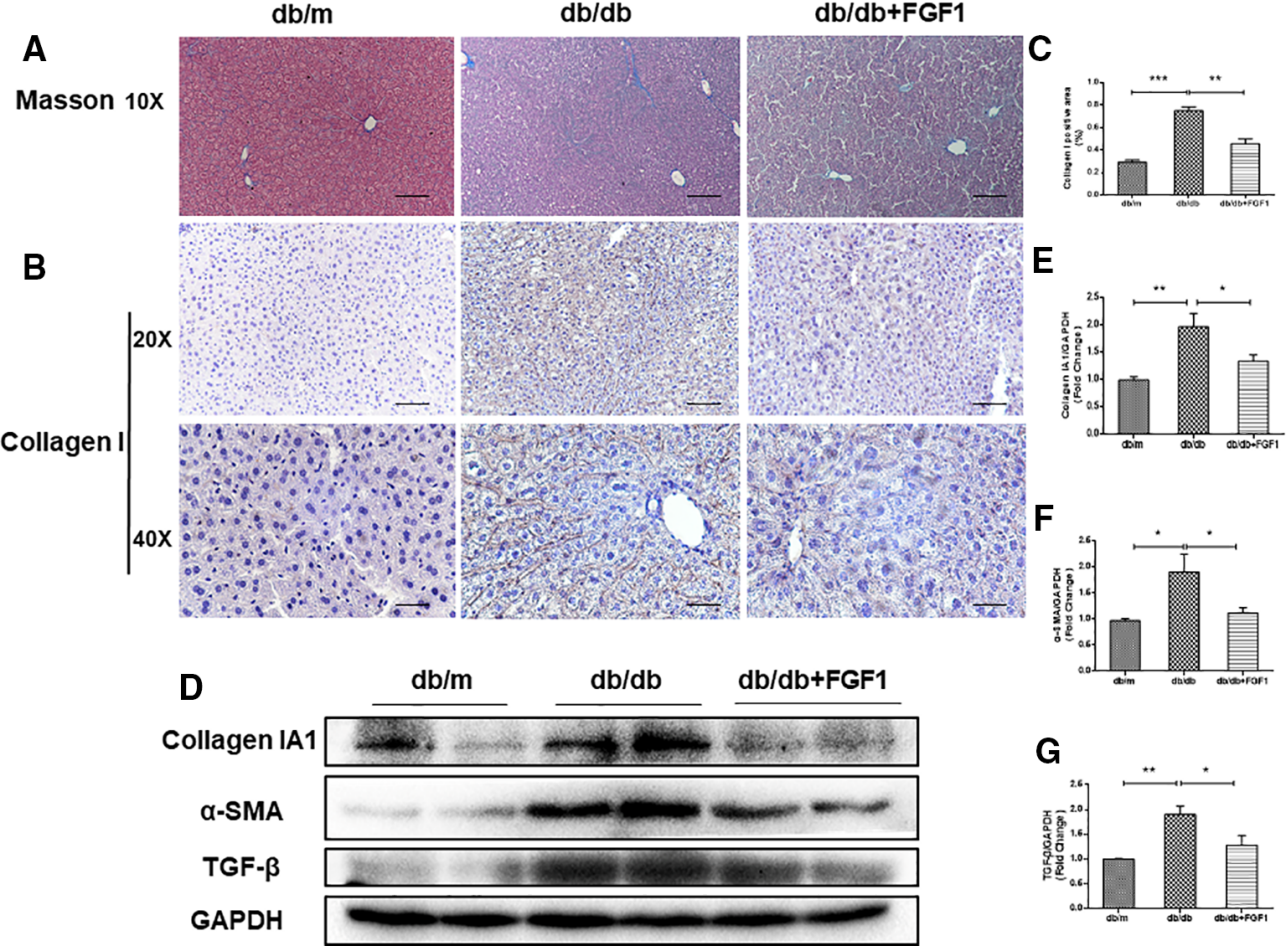
FGF1 treatment ameliorated diabetes-induced liver fibrosis by reducing deposition of collagen. **(A)** Masson staining of liver from db/m, db/db, and db/db + FGF1 mice (10 x:scale bars = 100 μm). **(B, C)** Immunohistochemical staining of collagen I and collagen I positive area of liver from db/m, db/db and db/db + FGF1 mice (20 x:scale bars = 50 μm 40 x:scale bars = 25 μm). **(D)** Protein expression of collagen I, α-SMA, and TGF-β in livers from db/m, db/db, and db/db + FGF1 mice. **(E–G)** Intensities of collagen I, α-SMA, and TGF-β normalized to GAPDH. All data are presented as mean ± SEM, n = 8. *P < 0.05 **P < 0.01 ***P < 0.001 *vs*. the db/m group and db/db + FGF1 group.

The expression of collagen is used as an index of liver injury. We graded the degree of hepatic fibrosis using Masson staining and collagen I immunohistochemical staining (**Figures 2A, B**). The areas of collagen fibers deposition were more significantly expressed in the central vein and surrounding sinus gaps in db/db mice than those in db/m control mice (**Figures 2C**). Consistent with the immunohistochemical results, western blotting results showed that protein levels of collagen IA1 in livers from db/db mice were significantly greater, but that were much lower in the FGF1 treatment group (**Figures 2D, E**). Furthermore, protein levels of α‐SMA and TGF-β were substantially greater in db/db mice livers (**Figures 2D, F, G**). All these changes in db/db mice were reversed by FGF1 treatment, suggesting that FGF1 inhibited diabetes-induced liver fibrosis by abolishing collagen accumulation in the liver.

### FGF1 Treatment Ameliorated Diabetes-Induced Hepatic Apoptosis

**Figure 3:**
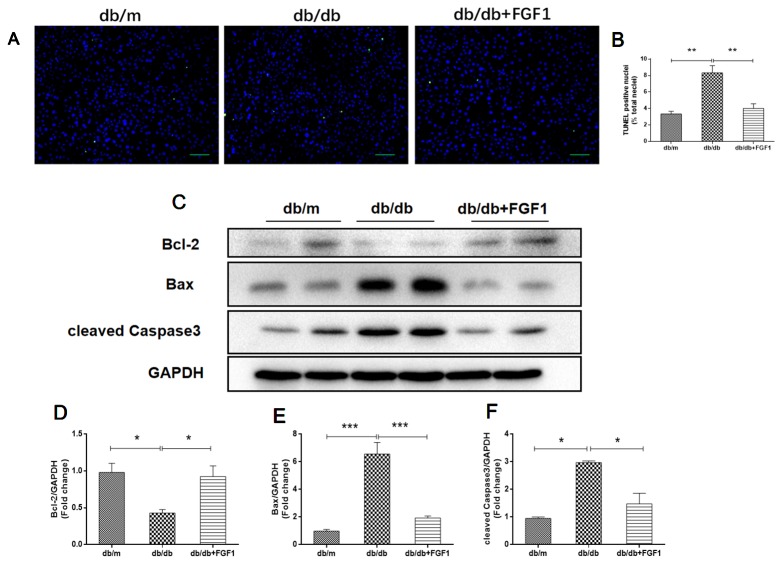
FGF1 ameliorated diabetes-induced hepatic apoptosis in liver. **(A, B)** TUNEL-positive nuclear staining of liver from db/m, db/db and db/db + FGF1 mice (scale bars = 100 μm). **(C)** Protein expression of cleaved caspase-3, Bax, and Bcl2 of liver from db/m, db/db, and db/db + FGF1 mice. **(D–F)** Intensities of cleaved caspase-3, Bax, and Bcl2 normalized to GAPDH. All data are presented as mean ± SEM, n = 8. *P < 0.05 **P < 0.01 ***P < 0.001 *vs*. the db/m group and db/db + FGF1 group.

To determine the mechanisms by which FGF1 protects against diabetes-induced liver damage, we examined apoptosis pathways. Compared with liver cells in db/m mice, we found that liver cells of db/db mice were severely damaged, characterized by irregular shapes and chaotic arrangement of cells. Numbers of TUNEL-positive staining cells were substantially greater in liver cells from db/db mice than those in db/m liver cells, an effect that was inhibited by FGF1 treatment (**Figures 3A, B**). Apoptosis indicators were also measured (**Figures 3C–F**). Expression levels of cleaved caspase-3 and Bax were much higher in db/db mice than those in db/m mice (**Figures 3C, E, F**). By contrast, compared with the db/m mice, Bcl2, an anti-apoptotic protein (Yu et al., 2019), was expressed at lower level in db/db mice livers (**Figures 3C, D**). All these changes were reversed by FGF1 treatment. Taken together, these observations suggest that FGF1 ameliorated diabetes-induced hepatic apoptosis in db/db mice.

### FGF1 Treatment Restored Defective Hepatic Autophagy in Diabetic Mice

**Figure 4:**
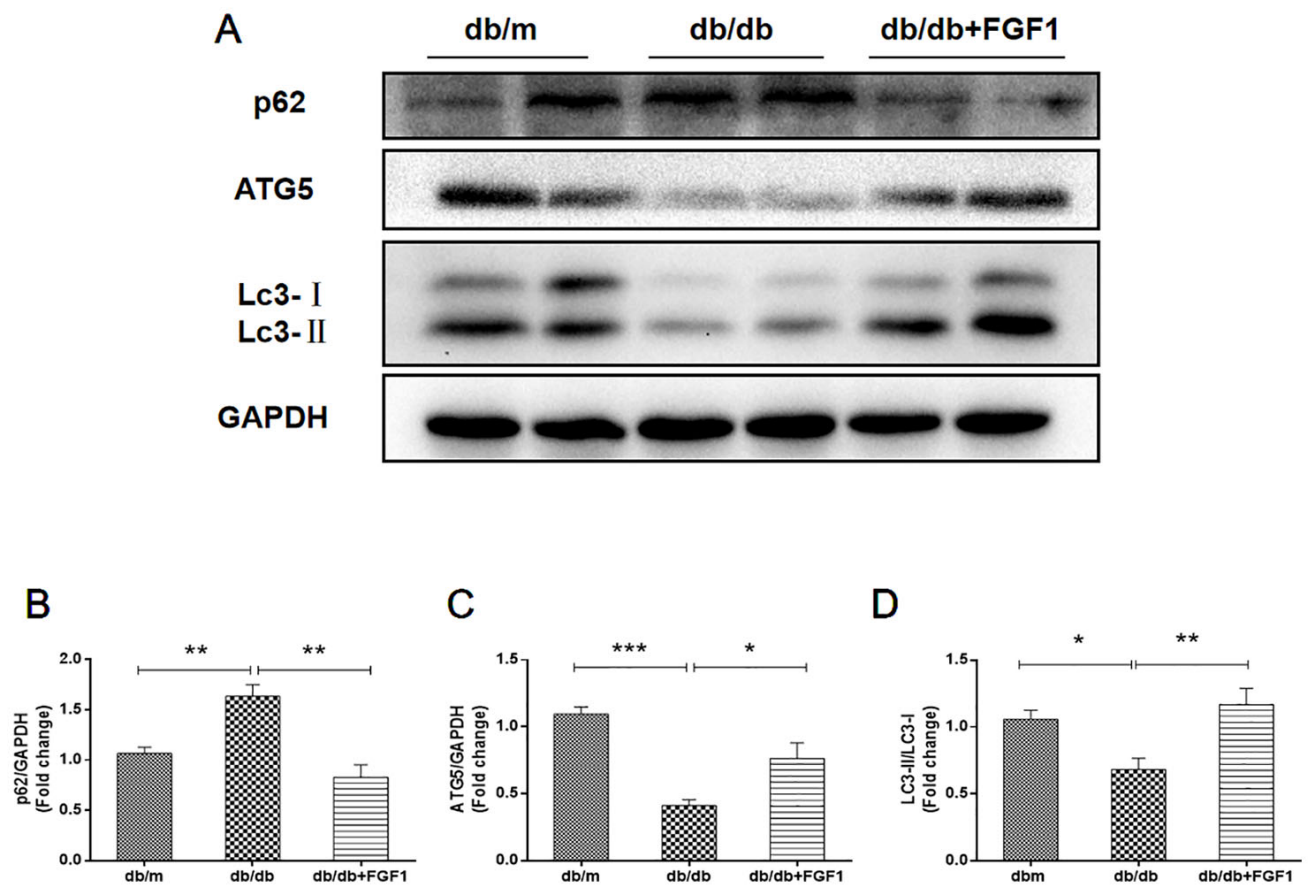
FGF1 restored hepatic autophagy in db/db mice. **(A)** Protein expression of p62, ATG5 and LC3 in livers from db/m, db/db and db/db + FGF1 mice. **(B, C)** Intensities of p62 and ATG5 normalized to GAPDH. **(D)** LC3II/LC3I. All data are presented as mean ± SEM, n = 8. *P < 0.05 **P < 0.01 ***P < 0.001 *vs*. the db/m group and db/db + FGF1 group.

Previous studies showed that diabetes impaired hepatic autophagy and that obesity caused markedly decreased autophagy in livers of both genetic and dietary mice models; this effect was demonstrated by decreased expression levels of LC3 and Atg5 (Yang et al., 2010). By contrast, p62 is involved in aggresome formation and is degraded through autophagy (Iwadate et al., 2014), with higher levels in ob/ob mice livers than in lean controls (Yang et al., 2010). To study the regulation of autophagy in the present diabetes model, we first examined expression patterns of molecular indicators of autophagy. In line with previous studies, we found that diabetes was characterized by lower expression of autophagy indicators in db/db mice livers, demonstrated by lower expression levels of LC3 and ATG5 proteins (**Figures 4A, C, D**). Levels of p62 protein level were substantially higher in db/db mice livers than that in db/m mice (**Figures 4A, B**). As expected, these changes were reversed by FGF1 treatment, suggesting that FGF1 treatment restored defective hepatic autophagy in db/db mice.

### FGF1 Treatment Blocked Diabetes-Induced Oxidative Stress in Liver

**Figure 5:**
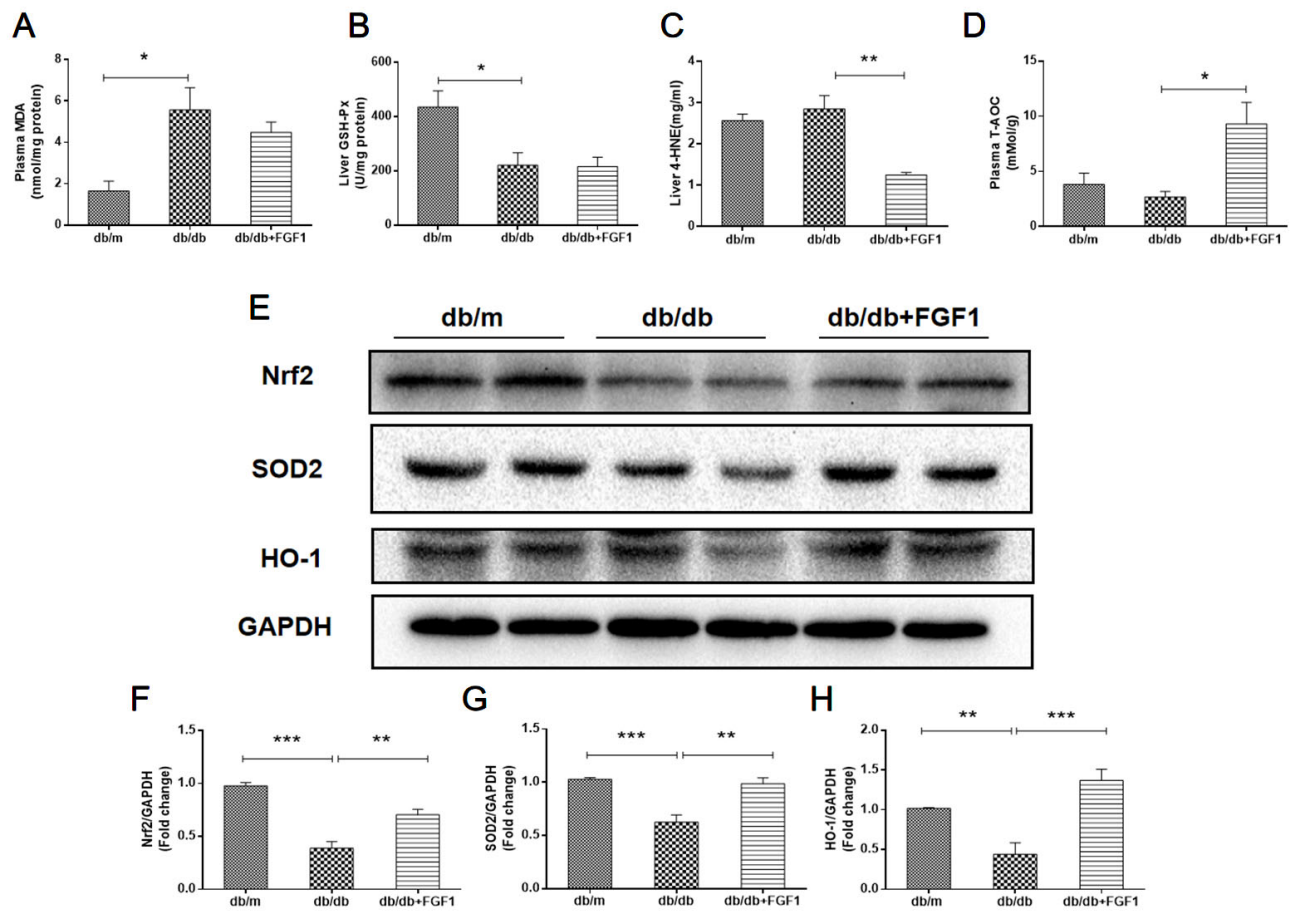
FGF1 blocked diabetes-induced liver oxidative stress. **(A)** Plasma malondialdehyde (MDA) levels. **(B)** Liver glutathione peroxidase (GSH-PX) levels. **(C)** liver 4-hydroxynonenal (4-HNE) levels. **(D)** Plasma total antioxidant capacity (T-AOC) levels. **(E)** Protein expression of Nrf2, HO-1, and SOD2 in livers from db/m, db/db, and db/db + FGF1 mice. **(F–H)** Intensities of Nrf2, HO-1, and SOD2 normalized to GAPDH. All data are presented as mean ± SEM, n = 8. *P < 0.05 **P < 0.01 ***P < 0.001 *vs*. the db/m group and db/db + FGF1 group.

Oxidative stress is a molecular mechanism that characterizes diabetes complications (Yang et al., 2015). Fatty acids in the liver induce free radical formation, causing lipid peroxidation and inducing release of proinflammatory cytokines. The release of malondialdehyde and 4-hydroxynonenal correspondingly causes cell death, protein cross-linkage, and stellate cells activation, leading to collagen synthesis and fibrosis (Xiao et al., 2017). Oxidative stress is also a critical factor in the progression of nonalcoholic fatty liver disease associated with diabetes (Mohamed et al., 2016). In the present study, we determined whether FGF1 treatment reversed diabetes-induced oxidative stress by measuring glutathione levels and oxidative stress markers (**Figure 5**). Plasma malondialdehyde (MDA) levels were significantly greater in db/db mice livers than those in of db/m mice livers (**Figure 5A**), an effect that was reduced by FGF1 treatment. As expected, liver glutathione peroxidase (GSH-PX) levels were lower in db/db mice livers than those in db/m mice livers, an effect that was increased by FGF1 treatment (**Figure 5B**). There were no significant differences in plasma MDA levels and liver GSH-PX levels between db/db mice and db/db + FGF1 mice. 4-hydroxynonenal (4-HNE)-protein adducts and protein carbonyl content were measured to assess lipid peroxidation and protein oxidation levels, respectively (Valle et al., 2012). Consistently higher levels of carbonyl and 4-HNE adducts were observed in db/db mice, an effect that was decreased by FGF1 treatment (**Figure 5C**). Although no significant differences were found between db/m mice and db/db mice with respect to liver 4-HNE levels and plasma T-AOC levels, FGF1 treatment significantly increased liver 4-HNE levels and plasma T-AOC levels in db/db mice (**Figures 5C, D**). HO-1 is the rate limiting enzyme in heme degradation, being the first step, catalyzing degradation of pro-oxidant heme to carbon monoxide, biliverdin, and ferrous iron (Abraham and Kappas, 2008). HO-1 gene expression is regulated by the transcriptional activator Nrf2 (Jiying et al., 2004; Igarashi and Sun, 2006). In the present study, FGF1 treatment caused significant increases in expression levels of Nrf2 in db/db mice, which ameliorated diabetes-induced oxidative stress in liver tissue (**Figures 5E, F**). Furthermore, expression levels of HO-1 in db/db mice liver were also lower than those of db/m mice, an effect that was diminished by FGF1 treatment (**Figures 5E, G**). SOD2 protein expression levels in liver were also significantly lower in db/db mice than those in db/m mice, an effect that was markedly ameliorated in FGF1-treated db/db mice (**Figures 5E, H**). Taken together, these data suggest that FGF1 treatment markedly reversed diabetes-induced hepatic oxidative stress in db/db mice.

In addition, we also examined FGFR1-AMP-activated protein kinase (AMPK) signaling pathway. Western blotting results show that FGFR1 expression level in db/db + FGF1 liver was markedly higher than that in db/db liver, indicating that FGFR1 signaling was activated by FGF1 treatment (**Supplement Figures 1A, B**). AMPK, a serine kinase, has been reported to enhance cellular antioxidant capacity through inducing activation of Nrf2 and HO-1 (Lee et al., 2019). In the present study, as shown in **Supplement Figures 1A, C**, hepatic P-AMPK levels were significantly lower in db/db mice compared with the db/m mice. As expected, these changes were reversed by FGF1 treatment. Taken together, these data suggest that FGF1 treatment markedly reversed diabetes-induced hepatic oxidative stress by regulating FGFR1-AMPK pathway.

### FGF1 Treatment Suppressed Diabetes-Induced Endoplasmic Reticulum Stress in Liver

**Figure 6:**
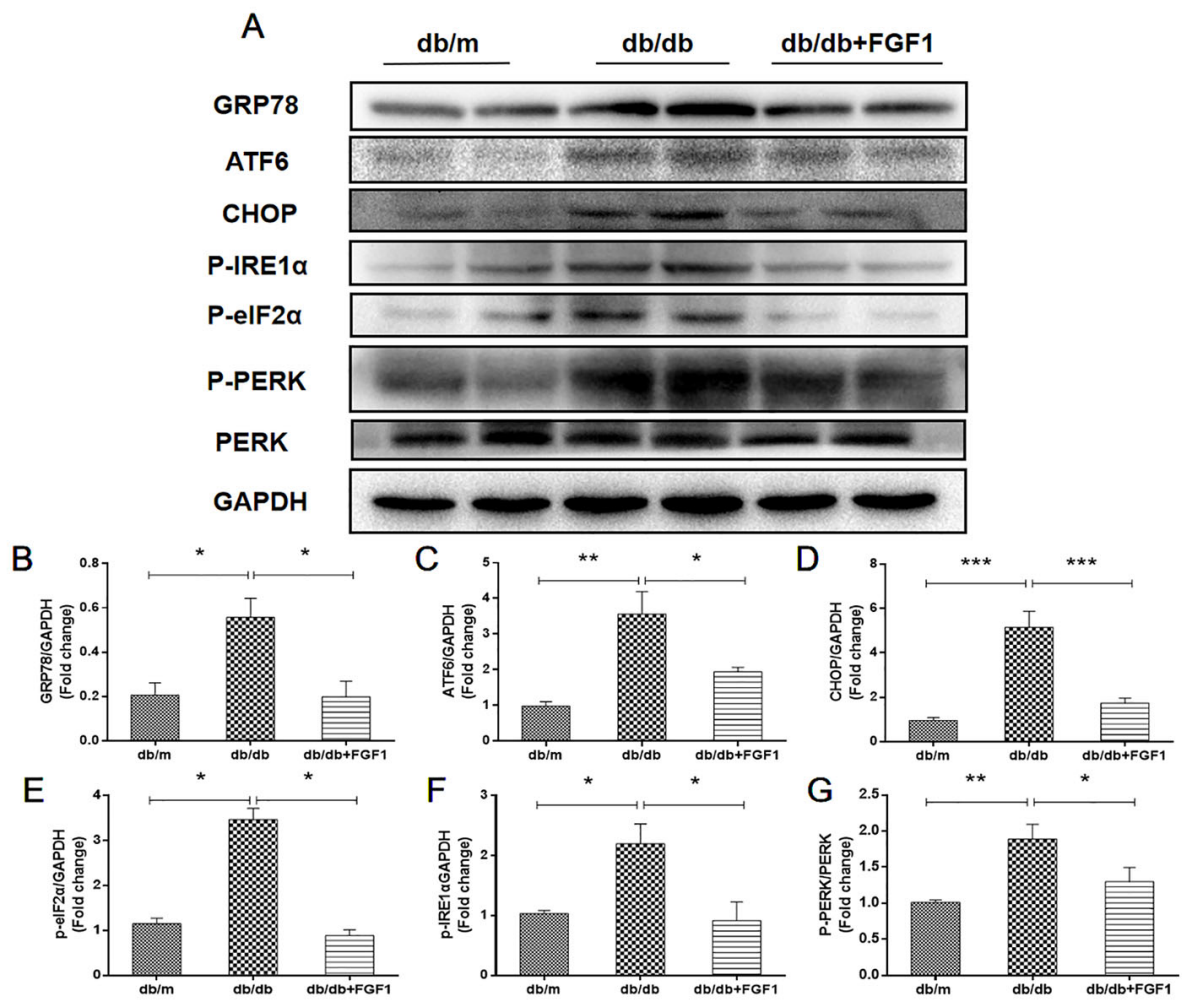
FGF1 suppressed diabetes-induced liver endoplasmic reticulum stress. **(A)** Protein expression of GRP78, ATF6, CHOP, P-eIF2α, P-IRE1α, and P-PERK in liver from db/m, db/db, and db/db + FGF1 mice. **(B–F)** Intensities of GRP78, ATF6, CHOP, P-eIF2α, and P-IRE1α normalized to GAPDH. **(G)** Intensity of P-PERK normalized to PERK. All data are presented as mean ± SEM, n = 8. *P < 0.05 **P < 0.01 ***P < 0.001 *vs*. the db/m group and db/db + FGF1 group.

Diabetes-induced liver injury causes changes in microstructure and morphology of liver tissues, leading to changes of hydrophilic and hydrophobic domains in endoplasmic reticulum, an effect that was closely associated with changes of cellular endoplasmic reticulum (ER) polarity. These data suggest that differences in ER polarity can suggest degree of diabetes-induced liver injury (Xiao et al., 2017). Proteins enter the ER as unfolded polypeptide chains, also called the unfolded protein response (UPR) (Ron and Walter, 2007). Elevation in the phosphorylation of eIF2α and PERK (P-eIF2α and P-PERK) is indicative marker of ER stress in diabetes mice. Furthermore, P-IRE1α, an early and crucial indicator of ER stress, plays an important role in mediating the UPR response under ER stress (Wang et al., 2013). To measure FGF1 treatment mediated inhibition of diabetes-induced ER stress in liver, we measured expression levels of ER stress protein markers. Hepatic protein levels of glucose-regulated protein 78 (GRP78), activating transcription factor 6 (ATF6), and C/EBP‐homologous protein (CHOP) were significantly greater in db/db mice and these changes were reversed by FGF1 treatment (**Figures 6A–D**). Notably, we identified dramatically higher P-IRE1α, P-eIF2α, and P-PERK protein expression levels in livers of db/db mice than in those of db/m mice, an effect that was inhibited by FGF1 treatment (**Figures 6A, E–G**). Taken together, these data suggest that FGF1 treatment significantly suppressed diabetes-induced ER stress.

## Discussion

In the present study, we demonstrated the glucose-lowing effect of FGF1 in db/db mice and its effects on the progression of liver injury in T2DM. These observations were associated with reduced plasma ALT, liver, and plasma TG levels, and decreased ER stress and oxidative stress as well as hepatic apoptosis. Furthermore, hepatic autophagy was corrected by FGF1 treatment.

The db/db mouse model is characterized by leptin receptor deficiency, and displays hyperinsulinemia, hyperglycemia, and glucosuria phenotypes (Razzoli et al., 2015). Previous studies have shown that FGF1 has therapeutic potential for insulin resistance and type 2 diabetes. A single injection of 0.5 mg/kg rFGF1 corrected hyperglycemia in ob/ob mice. This effect of FGF1 is dose-dependent, and even at the maximal dose (2.0 mg/kg) it does not produce hypoglycemia. Moreover, FGF1 has no effect on blood glucose or insulin levels in normoglycemic chow-fed mice. Furthermore, chronic rFGF1 treatment in mice with diet-induced obesity (DIO) also caused pronounced and sustained lowering of blood glucose levels and increased insulin sensitization (JaeMyoung et al., 2014). The glucose-lowering effects of FGF1 are mediated predominantly *via* FGF receptor 1 signaling. Injection of agonistic anti-FGF receptor 1 antibodies into obese diabetic mice induced acute and sustained amelioration of hyperglycemia, accompanied by significant improvement in hyperlipidemia, hyperinsulinemia, and hepatosteatosis (Wu et al., 2015b). In the present study, treatment of FGF1 for 4 weeks significantly reduced glucose levels in the circulation. Our results further confirmed the therapeutic potential of FGF1 for insulin resistance and type 2 diabetes.

Previous studies have shown that recombinant FGF1 (rFGF1) effectively improved hepatic damage and inflammation in ob/ob mice and choline-deficient mice, two etiologically-different models of NAFLD. Studies have reported that rFGF1 reduced steatosis in the periportal zone first, and subsequently improved hepatic lipid catabolism (Liu Y. et al., 2016). Consistent with these findings, we confirmed that FGF1 administration ameliorated diabetes-induced liver damage. We showed that FGF1 treatment reduced plasma ALT levels, ameliorated liver lipid accumulation as well as hepatic apoptosis and liver fibrosis and corrected hepatic autophagy. Furthermore, we demonstrated that FGF1 treatment blocked diabetes-associated collagen accumulation. All these findings are consistent with previous observations demonstrating the hepatic-protective effects of FGF1 in various animal models. Mechanistic studies demonstrated that induction of cellular stress in diabetic liver was inhibited by FGF1 treatment, suggesting that reduction of cellular stress was a potential molecular mechanism in course of FGF1 treatment for diabetes-induced liver injury.

Previous studies reported that FGF1 inhibited oxidative stress and consequently blocked diabetes-induced cardiomyopathy (Wu et al., 2016). Elevated oxidative stress is a major causal factor in diabetes-associated complications (Wu et al., 2011). Liver tissues from diabetic rats also showed significantly increased levels of reactive oxygen species (ROS), and dramatically lower levels of GSH, catalase, and superoxide dismutase (SOD) (Borderud et al., 2014). In the resting state, Nrf2 is in a non-free and non-active state of continuous degradation. When stimulated by electrophiles or ROS, Keap1 is uncoupled from Nrf2, allowing Nrf2 to transfer to the nucleus, and the Maf protein in the gene. Binding to the heterodimer recognizes and binds ARE, initiates transcription of the downstream protective protein genes, and enhances cell's ability to resist oxidative stress (McMahon et al., 2006). Nrf2 signaling may compensatorily increase the response to acute stress (Stewart et al., 2003), modulating expression of several oxidative damage genes, including HO-1 and SOD2 that are protective in diverse models of liver diseases (Ge et al., 2015; Peng et al., 2018). In our study, diabetes markedly resulted in down-regulation of Nrf2, HO-1 and SOD2, an effect that was reversed by FGF1 treatment. Furthermore, FGF1 treatment regulated FGFR1-AMPK signaling pathway. The above-mentioned data suggest that FGF1 treatment ameliorated hyperglycemia and subsequently oxidative stress during diabetes-induced liver injury *via* regulation of the FGFR1-AMPK-Nrf2/HO-1 signaling pathway.

A previous report showed that ER stress induced the production of reactive oxygen species (ROS) (Peng et al., 2018), thereby leading to oxidative stress (Hyung-Ryong et al., 2009). ER stress occurs when misfolded proteins accumulate in the ER lumen, and this is referred to as the unfolded protein response (UPR) (Hyung-Ryong et al., 2009). The UPR is made up of three main branches, controlled by ER membrane proteins: activating transcription factor (ATF), inositol-requiring enzyme (IRE), and protein kinase-like ER kinase (PERK), all of which are suppressed by binding to GRP78 (Hummasti and Hotamisligil, 2010). Elevated ER chaperone proteins are biological indicators of ER stress. In mammalian cells, 2 kinases, the inositol-requiring enzyme-1α (IRE1α) and PERK are responsible for activation of UPR. In diabetes, ER stress is caused by increased secretory demands to compensate for insulin resistance and is further aggravated by a fatty acid-induced ER dysfunction in β-cells (Saltiel and Kahn, 2001). Under these conditions, IRE1α activation induces messenger RNA (mRNA) splicing of transcription factors, whereas PERK activation inhibits protein translation and increases the expression of proapoptotic C/EBP-homologous protein (CHOP) *via* phosphorylation of eukaryotic initiation factor 2α (eIF2α) (Zinszner et al., 1998). In accordance with these data, we found that expression levels of ER stress markers, including CHOP, GRP78, and ATF6, were elevated in db/db mice. Protein expression levels of P**-**IRE1α, P-eIF2α, and P-PERK were much higher in livers of db/db mice than in those of db/m mice, an effect reversed by FGF1 treatment. Taken together, these data suggest that FGF1 exerted effects on diabetes-induced liver injury by suppressing diabetes-induced ER stress.

According to previous studies, overload of misfolded and/or aggregated proteins in ER lumen leads to ER dysfunction, resulting in ER stress and apoptosis (Varadarajan et al., 2012). Apoptosis is a primary characteristic of the pathogenesis of liver disease. In accordance with results of previous studies, we found that diabetes boosted apoptosis-related proteins expression levels including Bax and cleaved caspase3, and decreased Bcl2 protein expression level, all of which were reversed by FGF1 treatment. Hepatic apoptosis is regulated by autophagic activity. In addition, cross-talk between autophagy and apoptosis have been manifested by regulatory genes sharing common pathways. These regulatory genes include ATG5 and Bcl2 (Kewei, 2015). Previous studies showed that reduction in expression of autophagy-related protein ATG5, and ATG protein is an essential mediator of the autophagic process, resulting in defective insulin responsiveness. In the current study, we demonstrated that FGF1 increased defective hepatic autophagy in db/db mice *via* regulation of autophagy related proteins, including LC3II, ATG5 and p62 (**Figure 4**).

**Figure 7:**
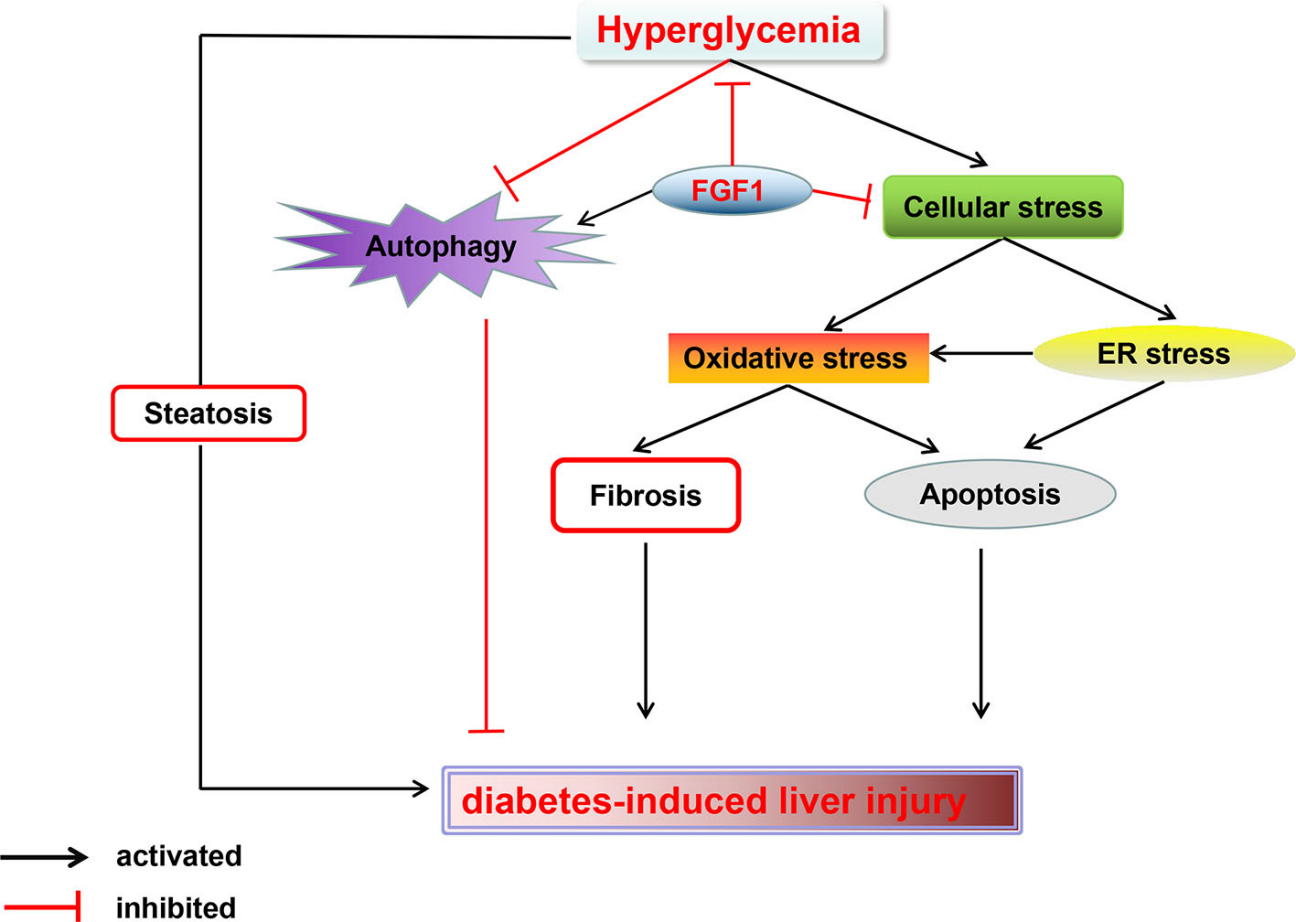
A Schematic showing the effects of FGF1 treatment on diabetic-induced liver injury. As reported that FGF1 can restore blood glucose levels to the normal range in reference 7. FGF1 treatment blocked hyperglycemia-induced cellular stress (oxidative stress and ER stress) and restored autophagy in liver, which ameliorates hepatic steatosis and fibrosis, consequently ameliorates diabetes-induced liver injury.

In summary, our findings expand the notion that FGF1 controlled hyperglycemia and normalized blood glucose levels in db/db mice. Treatment with FGF1 for 4 weeks significantly reduced blood glucose levels. These observations were associated with reduced diabetic liver damage. The protective effect of FGF1 on diabetes-induced injury was demonstrated by significantly lower plasma ALT activity levels, TG levels, and hepatic lipid accumulation as well as suppressed cellular stress, reduced diabetes-induced hepatic apoptosis, and restored defective hepatic autophagy. We confirmed that inhibition of cellular stress and restoring autophagy are the potential molecular mechanisms of FGF1 inhibition of diabetes-induced liver injury (**Figure 7**). One limitation is that we did not record food intake; this will be measured in a future study to fully understand the role of FGF1 in reducing glucose effect and ameliorating diabetes-induced liver injury.

## Data Availability Statement

The raw data supporting the conclusions of this article will be made available by the authors, without undue reservation, to any qualified researcher.

## Ethics Statement

The animal study was reviewed and approved by the Laboratory Animal Ethics Committee of Wenzhou Medical University & the Laboratory Animal Centre of Wenzhou Medical University.

## Author Contributions

YAL and JX conceived and designed the research. ZX performed the experiments and wrote the paper. YW gave important and thoughtful advices and performed the experiments. FW performed the statistical analysis and supplied a lot of technology and served as a fund assistant. XL, PW, YUL, JW, YIL, TJ, XP, XZ, and LX provided assistance with the experiments. All authors discussed the drafting of the manuscript.

## Funding

This work was partly supported by the National Natural Science Foundation of China (81300311), the Opening Project of Zhejiang Provincial Top Key Discipline of Pharmaceutical Sciences, the Technology Support Project of Xinjiang (2017E0267), the Xinjiang Tianshan Youth Project for Outstanding Young Scientists (2017Q007), Natural Science Foundation of Xinjiang Province (2018D01C228), Zhejiang public welfare technology research project (LGF19H030008), Ningbo Huimin project (2016C51004), and Ningbo Natural Science Funding (2018A610376), Project of Medical Technology of Zhejiang Province (2020KY908), and Natural Science Foundation of Xinjiang Uyghur Autonomous Region (2018D01C228).

## Conflict of Interest

The authors declare that the research was conducted in the absence of any commercial or financial relationships that could be construed as a potential conflict of interest.

## Supplementary Material

Supplement Figure 1 FGFR1-AMPK signaling pathway was activated. **(A)** Protein expression of FGFR1, P-AMPK and AMPK in liver from db/m, db/db and db/db + FGF1 mice. **(B)** Intensities of FGFR1 normalized to GAPDH. **(C)** Intensity of P-AMPK normalized to AMPK. All data are presented as mean ± SEM, n = 8. *P < 0.05 **P < 0.01 ***P < 0.001 *vs*. the db/m group and db/db + FGF1 group.

## References

[B1] Abraham N. G. Kappas A. (2008). Pharmacological and clinical aspects of heme oxygenase. Pharmacol. Rev. 60 (1), 79–127. 10.1124/pr.107.07104 18323402

[B2] Borderud S. P. Li Y. Burkhalter J. E. Sheffer C. E. Ostroff J. S. (2014). Electronic cigarette use among patients with cancer: characteristics of electronic cigarette users and their smoking cessation outcomes. Cancer 120 (22), 3527–3535. 10.1002/cncr.28811 25252116 PMC5642904

[B3] Guang L. LinTao S. Zilu C. Yuanyuan Q. Junjun X. Longwei Z. (2018). Fibroblast growth factor 1 ameliorates diabetic nephropathy by an anti-inflammatory mechanism. Kidney Int. 93 (1), 95–109. 10.1016/j.kint.2017.05.013 28750927 PMC5818994

[B4] Ge M. Yao W. Wang Y. Yuan D. Chi X. Luo G. (2015). Propofol alleviates liver oxidative stress via activating Nrf2 pathway. J. Surg. Res. 196 (2), 373–381. 10.1016/j.jss.2015.03.016 25890433

[B5] Gezginci-Oktayoglu S. Basaraner H. Yanardag R. Bolkent S. (2009). The effects of combined treatment of antioxidants on the liver injury in STZ diabetic rats. Dig. Dis. Sci. 54 (3), 538–546. 10.1007/s10620-008-0381-0 18712602

[B6] Hao L. S. Zhang X. L. An J. Y. Karlin J. Tian X. P. Dun Z. N. (2009). PTEN expression is down-regulated in liver tissues of rats with hepatic fibrosis induced by biliary stenosis. APMIS 117 (9), 681–691. 10.1111/j.1600-0463.2009.02515.x 19703128

[B7] Hyung-Ryong K. Geum-Hwa L. Eun Yi C. Soo-Wan C. Taeho A. Han-Jung C. (2009). Bax inhibitor 1 regulates ER-stress-induced ROS accumulation through the regulation of cytochrome P450 2E1. J. Cell Sci. 122 (null), 1126–1133. 10.1242/jcs.038430 19339548

[B8] Hummasti S. Hotamisligil G. S. (2010). Endoplasmic reticulum stress and inflammation in obesity and diabetes. Circ. Res. 107 (5), 579–591. 10.1161/CIRCRESAHA.110.225698 20814028

[B9] Igarashi K. Sun J. (2006). The heme-Bach1 pathway in the regulation of oxidative stress response and erythroid differentiation. Antioxid.s Redox Signaling 8 (null), 107–118. 10.1089/ars.2006.8.107 16487043

[B10] Iwadate R. Inoue J. Tsuda H. Takano M. Furuya K. Hirasawa A. (2014). High expression of SQSTM1/p62 protein is associated with poor prognosis in epithelial ovarian cancer. Acta Histochem. Cytochem. 47 (6), 295–301. 10.1267/ahc.14048 25859063 PMC4387266

[B11] Jiying S. Marjorie B. Yukari Z. Satoshi T. Mark G. Kazuhiko I. (2004). Heme regulates the dynamic exchange of Bach1 and NF-E2-related factors in the Maf transcription factor network. Proc. Natl. Acad. Sci. U. S. A. 101 (6), 1461–1466. 10.1073/pnas.0308083100 14747657 PMC341742

[B12] Jiang W. Jingjing Z. Chaochao H. Zecong X. Jingjing Y. Yi L. (2016). Comparative study of heparin-poloxamer hydrogel modified bFGF and aFGF for *in vivo* wound healing efficiency. ACS Appl. Mater. Interfaces 8 (29), 18710–18721. 10.1021/acsami.6b06047 27384134

[B13] JaeMyoung S. JohanW J. Maryam A. Regina G. Denise L. Olivia O. (2014). Endocrinization of FGF1 produces a neomorphic and potent insulin sensitizer. Nature 513 (7518), 436–439. 10.1038/nature13540 25043058 PMC4184286

[B14] Kewei W. (2015). Autophagy and apoptosis in liver injury. Cell Cycle (Georgetown Tex.) 14 (11), 1631–1642. 10.1080/15384101.2015.1038685 PMC4614283 25927598

[B15] Kasuga M. (2006). Insulin resistance and pancreatic beta cell failure. J. Clin. Invest. 116 (7), 1756–1760. 10.1172/JCI29189 16823472 PMC1483164

[B16] Lee E. H. Baek S. Y. Park J. Y. Kim Y. W. (2019). Rifampicin activates AMPK and alleviates oxidative stress in the liver as mediated with Nrf2 signaling. Chem. Biol. Interact. 315, 108889. 10.1016/j.cbi.2019.108889 31678598

[B17] Li X. (2019). The FGF metabolic axis. Front. Med. 13 (5), 511–530. 10.1007/s11684-019-0711-y 31495905 PMC7102389

[B18] Liu W. Struik D. Nies V. J. Jurdzinski A. Harkema L. de Bruin A. (2016). Effective treatment of steatosis and steatohepatitis by fibroblast growth factor 1 in mouse models of nonalcoholic fatty liver disease. Proc. Natl. Acad. Sci. U. S. A 113 (8), 2288–2293. 10.1073/pnas.1525093113 26858440 PMC4776526

[B19] Liu Y. Zhao C. Xiao J. Liu L. Zhang M. Wang C. (2016). Fibroblast growth factor 21 deficiency exacerbates chronic alcohol-induced hepatic steatosis and injury. Sci. Rep. 6, 31026. 10.1038/srep31026 27498701 PMC4976373

[B20] Marchesini G. Brizi M. Bianchi G. Tomassetti S. Bugianesi E. Lenzi M. (2001). Nonalcoholic fatty liver disease: a feature of the metabolic syndrome. Diabetes 50 (8), 1844–1850. 10.2337/diabetes.50.8.1844 11473047

[B21] McMahon M. Thomas N. Itoh K. Yamamoto M. Hayes J. D. (2006). Dimerization of substrate adaptors can facilitate cullin-mediated ubiquitylation of proteins by a “tethering” mechanism: a two-site interaction model for the Nrf2-Keap1 complex. J. Biol. Chem. 281 (34), 24756–24768. 10.1074/jbc.M601119200 16790436

[B22] Mohamed J. Nazratun Nafizah A. H. Zariyantey A. H. Budin S. B. (2016). Mechanisms of diabetes-induced liver damage: the role of oxidative stress and inflammation. Sultan Qaboos Univ. Med. J. 16 (2), e132–e141. 10.18295/squmj.2016.16.02.002 27226903 PMC4868511

[B23] Peng X. Dai C. Liu Q. Li J. Qiu J. (2018). Curcumin attenuates on carbon tetrachloride-induced acute liver injury in mice via modulation of the Nrf2/HO-1 and TGF-beta1/Smad3 pathway. Molecules 23 (1), 8–9. 10.3390/molecules23010215 PMC6017508 29351226

[B24] Razzoli M. McCallum J. Gurney A. Engeland W. C. Bartolomucci A. (2015). Chronic stress aggravates glucose intolerance in leptin receptor-deficient (db/db) mice. Genes Nutr. 10 (3), 458. 10.1007/s12263-015-0458-2 25791744 PMC4366428

[B25] Ron D. Walter P. (2007). Signal integration in the endoplasmic reticulum unfolded protein response. Nat. Rev. Mol. Cell Biol. 8 (7), 519–529. 10.1038/nrm2199 17565364

[B26] Saltiel A. R. Kahn C. R. (2001). Insulin signalling and the regulation of glucose and lipid metabolism. Nature 414 (6865), 799–806. 10.1038/414799a 11742412

[B27] Stewart D. Killeen E. Naquin R. Alam S. Alam J. (2003). Degradation of transcription factor Nrf2 via the ubiquitin-proteasome pathway and stabilization by cadmium. J. Biol. Chem. 278 (4), 2396–2402. 10.1074/jbc.M209195200 12441344

[B28] Valle A. Catalan V. Rodriguez A. Rotellar F. Valenti V. Silva C. (2012). Identification of liver proteins altered by type 2 diabetes mellitus in obese subjects. Liver Int. 32 (6), 951–961. 10.1111/j.1478-3231.2012.02765.x 22340678

[B29] Varadarajan S. Bampton E. T. W. Smalley J. L. Tanaka S. K. Caves R. E. Butterworth M. (2012). A novel cellular stress response characterised by a rapid reorganisation of membranes of the endoplasmic reticulum. Cell Death Differentiation 19 (12), 1896–1907. 10.1038/cdd.2012.108 22955944 PMC3504701

[B30] Wang F. Reece E. A. Yang P. (2013). Superoxide dismutase 1 overexpression in mice abolishes maternal diabetes-induced endoplasmic reticulum stress in diabetic embryopathy. Am. J. Obstet. Gynecol. 209 (4), 345 e341–347. 10.1016/j.ajog.2013.06.037 23791840 PMC3786037

[B31] Wang X. Zhang X. Wang F. Pang L. Xu Z. Li X. (2019). FGF1 protects against APAP-induced hepatotoxicity via suppression of oxidative and endoplasmic reticulum stress. Clin. Res. Hepatol. Gastroenterol. 43 (6), 1–7. 10.1016/j.clinre.2019.03.006 31029643

[B32] Wu A. L. Kolumam G. Stawicki S. Chen Y. Li J. Zavala-Solorio J. (2011). Amelioration of type 2 diabetes by antibody-mediated activation of fibroblast growth factor receptor 1. Sci. Transl. Med. 3 (113), 113ra126. 10.1126/scitranslmed.3002669 22174314

[B33] Wu Y. Wang F. Fu M. Wang C. Quon M. J. Yang P. (2015a). Cellular stress, excessive apoptosis, and the effect of metformin in a mouse model of Type 2 diabetic embryopathy. Diabetes 64 (7), 2526–2536. 10.2337/db14-1683 25720389 PMC4477360

[B34] Wu Y. Wang F. Reece E. A. Yang P. (2015b). Curcumin ameliorates high glucose-induced neural tube defects by suppressing cellular stress and apoptosis. Am. J. Obstet. Gynecol. 212 (6), 802 e801–808. 10.1016/j.ajog.2015.01.017 25595578 PMC4457597

[B35] Wu Y. Reece E. A. Zhong J. Dong D. Shen W. B. Harman C. R. (2016). Type 2 diabetes mellitus induces congenital heart defects in murine embryos by increasing oxidative stress, endoplasmic reticulum stress, and apoptosis. Am. J. Obstet. Gynecol. 215 (3), 366 e361–366 e310. 10.1016/j.ajog.2016.03.036 27038779 PMC5260663

[B36] Wu Y. Li Y. Jiang T. Yuan Y. Li R. Xu Z. (2018). Reduction of cellular stress is essential for Fibroblast growth factor 1 treatment for diabetic nephropathy. J. Cell Mol. Med. 22 (12), 6294–6303. 10.1111/jcmm.13921 30320493 PMC6237604

[B37] Xiao H. Wu C. Li P. Gao W. Zhang W. Zhang W. (2017). Ratiometric photoacoustic imaging of endoplasmic reticulum polarity in injured liver tissues of diabetic mice. Chem. Sci. 8 (10), 7025–7030. 10.1039/C7SC02330H 29147529 PMC5642195

[B38] Yang L. Li P. Fu S. Calay E. S. Hotamisligil G. S. (2010). Defective hepatic autophagy in obesity promotes ER stress and causes insulin resistance. Cell Metab. 11 (6), 467–478. 10.1016/j.cmet.2010.04.005 20519119 PMC2881480

[B39] Yang P. Reece E. A. Wang F. Gabbay-Benziv R. (2015). Decoding the oxidative stress hypothesis in diabetic embryopathy through proapoptotic kinase signaling. Am. J. Obstet. Gynecol. 212 (5), 569–579. 10.1016/j.ajog.2014.11.036 25434839 PMC4417047

[B40] Yu L. Zheng J. Li J. Wang Y. Lu X. Fan X. (2019). Integrating serum exosomal microRNA and liver microRNA profiles disclose the function role of autophagy and mechanisms of Fructus Meliae Toosendan-induced hepatotoxicity in mice. BioMed. Pharmacother. 123, 109709. 10.1016/j.biopha.2019.109709 31855734

[B41] Zinszner H. Kuroda M. Wang X. Batchvarova N. Lightfoot R. T. Remotti H. (1998). CHOP is implicated in programmed cell death in response to impaired function of the endoplasmic reticulum. Genes Dev. 12 (7), 982–995. 10.1101/gad.12.7.982 9531536 PMC316680

