# Supplementary figures and images for: Fibroblast Growth Factor 1 Ameliorates Diabetes-Induced Liver Injury by Reducing Cellular Stress and Restoring Autophagy
Source: Front Pharmacol. 2020 Mar 3;11:52. doi: 10.3389/fphar.2020.00052 (PMC7062965; doi:10.3389/fphar.2020.00052)

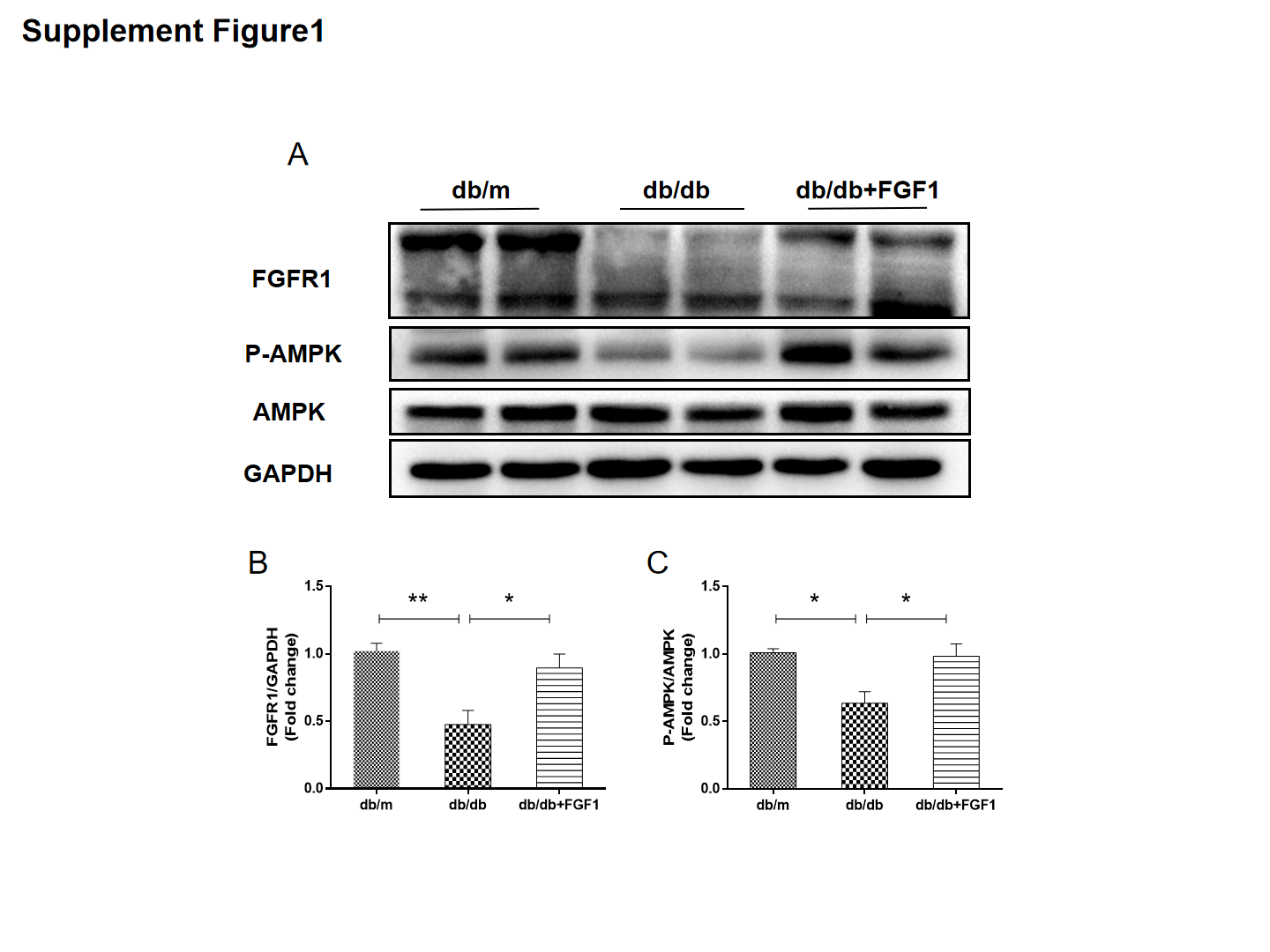

Supplement: Supplement Figure 1 — FGFR1-AMPK signaling pathway was activated. (A) Protein expression of FGFR1, P-AMPK and AMPK in liver from db/m, db/db and db/db + FGF1 mice. (B) Intensities of FGFR1 normalized to GAPDH. (C) Intensity of P-AMPK normalized to AMPK. All data are presented as mean ± SEM, n = 8. *P < 0.05 **P < 0.01 ***P < 0.001 vs. the db/m group and db/db + FGF1 group. [file Image_1.tif]
